# Commentary: Efficacy and safety of massage for postoperative stress in colorectal cancer patients: a randomized, controlled, three-arm trial

**DOI:** 10.3389/fonc.2025.1626131

**Published:** 2025-07-28

**Authors:** Yingjie Liu, Keying Wang, Wenjiang Wu

**Affiliations:** Shenzhen Hospital (Futian) of Guangzhou University of Chinese Medicine, Shenzhen, China

**Keywords:** massage, colorectal cancer, commentary, postoperative stress, heterogeneity

## 1 Introduction

While Werthmann et al. pioneer RE efficacy exploration in surgical oncology, critical methodological constraints necessitate scholarly scrutiny to advance non-pharmacological intervention research. The use of HRV as an objective stress marker aligns with contemporary psychoneuroimmunology frameworks linking autonomic nervous system function to clinical outcomes. However, the study’s execution reveals conceptual and technical gaps that warrant scholarly discourse to advance future trial design. While pandemic-related recruitment constraints (e.g., center expansion, visitor restrictions) introduced unavoidable challenges, fundamental design limitations warrant priority addressing.

## 2 Critical methodological challenges

### 2.1 HRV measurement validity compromised

**Figure 1:**
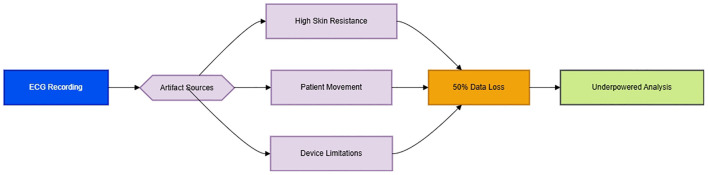
Pathophysiological cascade of ECG artifact generation [Original schematic by authors, based on artifact mechanisms described in (1)].

The substantial 50% data loss of primary outcome data as illustrated in **Figure 1** stems from unaddressed ECG artifacts reflects critical methodological limitations. Postoperative patients inherently exhibit elevated skin-electrode impedance from diaphoresis and restricted mobility, while surgical pain and nursing interventions inevitably induce motion artifacts—both entirely predictable yet conspicuously absent from mitigation protocols. Mitigating these artifacts requires ​integrated technical protocols: hydrogel electrodes to improve skin contact during diaphoresis, motion artifact compensation via accelerometer synchronization, and real-time artifact detection algorithms. The reliance on single-channel ECG without impedance monitoring or motion artifact algorithms reflects an archaic approach to perioperative autonomic monitoring, directly contravening modern standards for ambulatory HRV research (1). Such negligence irrevocably undermines the study’s internal validity, transforming the primary outcome into a speculative exercise rather than an evidence-based conclusion. To salvage scientific integrity, future trials must implement multimodal validation to ensure signal fidelity, with pilot validation to ensure clinical feasibility. Pilot validation of simplified configurations (e.g., hydrogel electrodes + inertial motion units) should precede complex implementations to ensure clinical feasibility in postoperative settings.

### 2.2 Control group selection paradox

Empathic conversation acts as an active neuromodulatory intervention rather than a passive control. To isolate RE-specific effects, Validated sham interventions should replace active controls, such as ​static tactile placebo​ (practitioner’s non-massaging hand placement) coupled with ​device-based deception​ (vibrating massager without therapeutic kneading), while standardizing verbal interactions using neutral scripts devoid of emotional engagement. As an active behavioral intervention with empirically demonstrated modulation of hypothalamic-pituitary-adrenal axis activity and vagal tone, it functions as a potent psychophysiological intervention rather than a neutral control. This aligns with evidence that even non-pharmacological interactions like empathic conversation exert significant neurobiological effects, invalidating their use as inert controls (2). This design inadvertently compares two active interventions—tactile-somatosensory modulation versus cognitive-emotional engagement—creating comparison bias as acknowledged by the authors (Section 5.2). The resultant Type II error risk is exacerbated by visitation restrictions may have enhanced receptiveness to conversational interventions. This control selection paradox contrasts with established placebo-control paradigms spanning three decades of placebo-control paradigms in manual therapy research, where validated sham techniques (e.g., non-therapeutic touch or deactivated devices) exist precisely to isolate biomechanical effects from contextual healing rituals. This fundamental misstep reduces the trial to an inconclusive comparison of two active treatments rather than a rigorous efficacy assessment.

### 2.3 Heterogeneity mismanagement

The statistically significant imbalance in tumor localization (*p*=0.02) and unaddressed gender disparities reveal a unaddressed heterogeneity in tumor localization and gender distribution that significantly biases stress responses. Rectal resection inherently provokes greater autonomic disruption through pelvic plexus dissection—a procedure absent in colon surgery—yet the study conflates these distinct entities, masking subgroup-specific treatment effects. Simultaneously, the 30% absolute difference in female representation between groups introduces unmeasured endocrine confounders, given estrogen’s established modulation of vagal activity and HPA axis function. This methodological oversimplification obscures biologically critical variations. Future studies must stratify cohorts by surgical stress levels, particularly isolating high-risk subgroups undergoing pelvic autonomic plexus dissection (e.g., rectal resection) from moderate-risk procedures (3). Sex-dependent autonomic responses are well-documented; for instance, Jarczok et al. (4) demonstrated that vagally-mediated HRV metrics exhibit clinically significant sex dimorphism, amplifying bias risks in heterogenous cohorts (5).

## 3 Novel perspectives for future research

### 3.1 Precision stress phenotyping

The imperative for multidimensional stress fingerprinting lies in transcending unitary biomarker approaches to capture neuroendocrine-immune-metabolic crosstalk. A clinically deployable biomarker panel should combine autonomic (HRV-SDNN), endocrine (salivary cortisol slope), and inflammatory (hs-CRP) indices. This multidimensional profiling can identify RE-responsive phenotypes, especially in patients exhibiting preoperative metabolic dysregulation or immune hyperactivation.

### 3.2 Mechanistic dose-response exploration

Dose-response optimization requires probing temporal dynamics through continuous HRV telemetry during variable-length RE sessions(5-20min). Establish minimal effective duration through dose escalation trials. Probing variable exposure windows (5–20 min) via continuous HRV telemetry will delineate the minimum effective dose for vagal activation and map response decay curves—essential for optimizing session economics and exploring potential response plateaus to optimize session economics in real-world settings where dose-response topography dictates clinical utility.

### 3.3 Patient-centered implementation

Building upon the authors’ recognition that ‘patient preferences and previous massage experiences’ significantly modulate outcomes (Section 5.2), future trials must embed quantitative phenotyping of tactile sensitivity and therapy receptiveness into stratification frameworks.

## 4 Discussion

Crucially, autonomic changes must translate to patient-important outcomes: ≥30% reduction in opioid consumption, accelerated return of bowel function (>12hr faster), and clinically meaningful anxiety reduction (HADS-A decrease≥4 points). Linking biomarkers to these endpoints will determine RE’s value in surgical rehabilitation (3). Mechanistic mediation analyses should quantify the proportion of clinical benefit attributable to autonomic changes (e.g., HRV-mediated opioid reduction), differentiating direct effects from biomarker-mediated pathways. Notably, the trial conclusively demonstrated RE’s safety profile – zero intervention-related complications among 68 patients align with historical safety data of manual therapies. This foundational safety evidence justifies further efficacy optimization in high-autonomic-risk cohorts. Null outcomes here mandate methodological recalibration: deploying artifact-resistant ambulatory monitoring to salvage >50% lost ECG data; phenotyping vulnerability in emergency surgery cohorts where stress pathways amplify; and triangulating SDNN shifts with patient-perceived recovery metrics via mixed-methods designs. Adaptive trials modulating RE dosage against biomarker trajectories could rescue the 9.12 ms SDNN signal from statistical oblivion, converting mechanistic noise into actionable precision rehabilitation.

## References

[1] Shaffer F Ginsberg JP . An overview of heart rate variability metrics and norms: Implications for ambulatory monitoring. Front Public Health. (2022) 10:258. doi: 10.3389/fpubh.2017.00258, PMID: 29034226 PMC5624990

[2] Colloca L Barsky AJ . Placebo and nocebo effects. N Engl J Med. (2020) 382:554–61. doi: 10.1056/NEJMra1907805, PMID: 32023375

[3] Joliat GR Kobayashi K Hasegawa K Thomson JE Padbury R Scott M . Guidelines for perioperative care for liver surgery: Enhanced Recovery After Surgery (ERAS) Society Recommendations 2022. World J Surg. (2023) 47:11–34. doi: 10.1007/s00268-022-06732-5, PMID: 36310325 PMC9726826

[4] Jarczok MN Koenig J Wittling A Fischer JE Thayer JF . First evaluation of an index of low vagally-mediated heart rate variability as a marker of health risks in human adults: Proof of Concept. J Clin Med. (2019) 8:1940. doi: 10.3390/jcm8111940, PMID: 31717972 PMC6912519

[5] Rohleder N . Stress and inflammation – The need to address the gap in the transition between acute and chronic stress effects. Psychoneuroendocrinology. (2020) 115:104589. doi: 10.1016/j.psyneuen.2019.02.021, PMID: 30826163

